# Supplementary material for: Geographical Distribution and Multimethod Species Identification of Forensically Important Necrophagous Flies on Hainan Island
Source: Insects. 2023 Nov 20;14(11):898. doi: 10.3390/insects14110898 (PMC10672153; doi:10.3390/insects14110898)
Supplement: Supplementary file 1 [file insects-14-00898-s001.zip › insects-2679222-supplementary.pdf]

Supplementary Data File

Table S1 Species composition of necrophagous flies in Hainan Island

Table S2 Interspecific genetic divergence among 30 species based on analyses of COI.

Table S1 Species composition of necrophagous flies in Hainan Island

Family	Subgenus	Species	Quantity of collected samples
Sarcophagidae (4,847)	*Parasarcophaga* (2,585)	*Sarcophaga dux* Thomson, 1869	562
		Sarcophaga misera Walker, 1849	1008
		Sarcophaga sericea (Robineau-Desvoidy, 1830)	700
		Sarcophaga scopariiformis Senior-White, 1927	5
		Sarcophaga albiceps Meigen, 1826	77
		Sarcophaga ruficornis (Fabricius, 1794)	52
		Sarcophaga brevicornis (Ho, 1934)	38
		Sarcophaga similis Meade, 1876	81
		Sarcophaga semenovi Rohdendorf, 1925	62
	Boettcherisca (1,392)	*Sarcophaga peregrina* (Robineau-Desvoidy 1830)	1346
		Sarcophaga formosensis (KirneretLopes,1961)	46
	Lioproctia (11)	*Sarcophaga pattoni* Senior-White, 1924	11
	Seniorwhitea (166)	*Sarcophaga reciproca* Walker, 1856	166
	Pierretia (15)	*Sarcophaga caudagalli* Bottcher, 1912	4
		Sarcophaga josephi Bottcher, 1912	11
	Leucomyia 630	*Sarcophaga cinerea* (Fabricius, 1794)	630
	Bercaea (48)	*Sarcophaga cruentata* Meigen, 1826	48
Calliphoridae (6,570)	*Chrysomya* (3,936)	*Chrysomya pinguis* (Walker,1858)	536
		Chrysomya megacephala (Fabricius, 1794)	3400
	Achoetandrus (1,331)	*Chrysomya rufifacies* (Macquart, 1843)	1131
		Chrysomya villeneuvi Patton, 1922	200
	Hemipyrellia (980)	*Hemipyrellia ligurriens* (Wiedemann,1830)	980
	Lucilia (323)	*Lucilia cuprina* (Wiedemann, 1830)	70
		Lucilia sericata (Meigen, 1826)	3
		Lucilia bazini Seguy, 1934	22
		Lucilia hainanensis Fan, 1965	23
		Lucilia porphyrina (Walker, 1856)	205
Muscidae (779)	*Ophyra* (194)	*Hydrotaea chalcogaster* (Wiedemann, 1824)	157
		Hydrotaea spinigera Hennig, 1962	37
	Musca (516)	*Musca domestica* Linnaeus, 1758	416
		Musca ventrosa Wiedemann, 1830	18
		Musca sorbens Wiedemann, 1830	82

	Synthesiomyia (65)	*Synthesiomyia nudiseta* (Wulp, 1883)	65
	Muscina (4)	*Muscina stabulans* (Fallen, 1817)	4
Fanniidae (3)	*Fannia* (3)	*Fannia pusio* (Wiedemann, 1830)	3
Anthomyiidae (52)	*Anthomyia* (52)	*Anthomyia illocata* Walker, 1856	52

Note: The number in bracket indicates the size of samples collected.

Table S2 Interspecific genetic divergence among 30 species based on analyses of COI

S. princeps																													
S. brevicornis	0.075																												
S. misera	0.091	0.058																											
S. caudagalli	0.095	0.077	0.085																										
S. pattoni	0.090	0.082	0.102	0.089																									
S. ruficornis	0.074	0.072	0.077	0.089	0.088																								
S. josephi	0.089	0.089	0.095	0.102	0.103	0.095																							
S. peregrina	0.094	0.070	0.073	0.083	0.089	0.081	0.104																						
S. dux	0.088	0.052	0.067	0.082	0.096	0.081	0.095	0.077																					
S. albiceps	0.085	0.061	0.048	0.086	0.100	0.077	0.096	0.073	0.071																				
S. scopariiformis	0.099	0.076	0.084	0.085	0.094	0.091	0.092	0.076	0.088	0.079																			
S. formosensis	0.095	0.073	0.068	0.092	0.092	0.083	0.102	0.028	0.081	0.072	0.075																		
S. cinerea	0.099	0.092	0.106	0.111	0.113	0.096	0.112	0.103	0.101	0.102	0.107	0.107																	
C. villeneuvi	0.109	0.096	0.106	0.115	0.119	0.105	0.115	0.111	0.105	0.102	0.107	0.113	0.125																
C. megacephala	0.108	0.105	0.113	0.125	0.125	0.105	0.122	0.122	0.109	0.109	0.118	0.122	0.132	0.064															
C. pinguis	0.112	0.108	0.120	0.128	0.125	0.107	0.130	0.125	0.114	0.110	0.125	0.123	0.136	0.071	0.025														
C. rufifacies	0.107	0.103	0.103	0.123	0.115	0.108	0.112	0.119	0.105	0.103	0.102	0.116	0.119	0.045	0.066	0.073													
L. hainanensis	0.116	0.105	0.108	0.123	0.119	0.099	0.121	0.112	0.115	0.105	0.117	0.109	0.127	0.094	0.097	0.096	0.099												
L. cuprina	0.105	0.102	0.103	0.109	0.118	0.100	0.119	0.111	0.105	0.097	0.109	0.113	0.119	0.095	0.081	0.089	0.098	0.061											
L. porphyrina	0.115	0.106	0.111	0.126	0.119	0.097	0.122	0.113	0.115	0.109	0.119	0.111	0.127	0.095	0.097	0.098	0.098	0.008	0.059										
L. bazini	0.111	0.100	0.105	0.118	0.114	0.103	0.111	0.105	0.102	0.099	0.115	0.112	0.116	0.089	0.090	0.094	0.095	0.063	0.065	0.065									
L. sericata	0.107	0.100	0.099	0.105	0.118	0.100	0.117	0.110	0.102	0.095	0.108	0.112	0.122	0.096	0.084	0.087	0.099	0.055	0.010	0.062	0.065								
H. ligurriens	0.109	0.100	0.102	0.119	0.118	0.107	0.123	0.120	0.109	0.105	0.119	0.120	0.128	0.094	0.085	0.093	0.099	0.072	0.062	0.073	0.076	0.064							
M. sorbens	0.119	0.119	0.128	0.126	0.122	0.115	0.126	0.125	0.115	0.115	0.127	0.128	0.144	0.117	0.102	0.099	0.117	0.112	0.105	0.114	0.112	0.103	0.109						

M. domestica	0.132	0.125	0.132	0.128	0.122	0.124	0.133	0.130	0.129	0.120	0.129	0.132	0.148	0.105	0.099	0.097	0.101	0.112	0.109	0.113	0.107	0.109	0.114	0.069					
H. chalcogaster	0.109	0.099	0.110	0.125	0.120	0.112	0.121	0.114	0.114	0.115	0.117	0.124	0.128	0.109	0.109	0.121	0.111	0.098	0.092	0.101	0.095	0.089	0.097	0.105	0.111				
H. spinigera	0.117	0.109	0.118	0.123	0.132	0.117	0.122	0.125	0.113	0.122	0.119	0.127	0.128	0.102	0.105	0.115	0.102	0.111	0.097	0.112	0.100	0.097	0.102	0.110	0.105	0.076			
M. stabulans	0.130	0.120	0.125	0.142	0.141	0.130	0.143	0.141	0.128	0.120	0.134	0.140	0.148	0.125	0.121	0.125	0.124	0.120	0.109	0.119	0.118	0.108	0.115	0.123	0.119	0.115	0.120		
S. nudiseta	0.114	0.116	0.121	0.135	0.139	0.119	0.138	0.139	0.129	0.124	0.128	0.142	0.138	0.120	0.119	0.129	0.112	0.120	0.112	0.122	0.115	0.109	0.109	0.121	0.122	0.097	0.100	0.125	
A. illocata	0.116	0.101	0.116	0.128	0.129	0.110	0.122	0.127	0.118	0.113	0.115	0.129	0.130	0.100	0.106	0.106	0.103	0.103	0.099	0.103	0.112	0.096	0.105	0.116	0.109	0.104	0.113	0.113	0.119
